# Case Report: Plasma Biomarkers Reflect Immune Mechanisms of Guillain–Barré Syndrome

**DOI:** 10.3389/fneur.2021.720794

**Published:** 2021-09-03

**Authors:** Chia-Lun Wu, Chung-Hao Chao, Shun-Wen Lin, Yu-Yi Chien, Wen-Yi Huang, Wei-Chieh Weng, Feng-Chieh Su, Yi-Chia Wei

**Affiliations:** ^1^Department of Neurology, Chang Gung Memorial Hospital, Keelung City, Taiwan; ^2^School of Medicine, Chang Gung University, Taoyuan City, Taiwan; ^3^Community Medicine Research Center, Chang Gung Memorial Hospital, Keelung City, Taiwan

**Keywords:** Guillain–Barré syndrome, cytokine, blood biomarker, Luminex, bead-based multiplexing immuno assay, immune mechanism, BDNF

## Abstract

This case series reported a group of patients with Guillain–Barré syndrome (GBS) and their plasma cytokine changes before and after immunotherapy. We aimed to understand GBS's pathogenesis and pathophysiology through observing the interval differences of the representative cytokines, which were the thymus and activation regulated chemokine (TARC) for T-cell chemotaxis, CD40 ligand (CD40L) for cosimulation of B and T cells, activated complement component C5/C5a, and brain-derived neurotrophic factor (BDNF) for survival and regenerative responses to nerve injuries. The fluorescence magnetic bead-based multiplexing immunoassay simultaneously quantified the five cytokines in a single sample. From June 2018 to December 2019, we enrolled five GBS patients who had completed before–after blood cytokine measurements. One patient was diagnosed with paraneoplastic GBS and excluded from the following cytokine analysis. The BDNF level decreased consistently in all the patients and made it a potential biomarker for the acute stage of GBS. Interval changes of the other four cytokines were relatively inconsistent and possibly related to interindividual differences in the immune response to GBS triggers, types of GBS variants, and classes of antiganglioside antibodies. In summary, utilizing the multiplexing immunoassay helps in understanding the complex immune mechanisms of GBS and the variation of immune responses in GBS subtypes; this method is feasible for identifying potential biomarkers of GBS.

## Introduction

### Immune Mechanism of Guillain–Barré Syndrome

Guillain–Barré syndrome (GBS) is an inflammatory disease of the peripheral nervous system induced by aberrant immune responses to preceding triggers. The pathogenesis of GBS is partly due to molecular mimicry of antecedent pathogens and subsequent provocation of generating cross-reactive antibodies that target different gangliosides in human peripheral nerves. Gangliosides are polymorphic sialic acid–containing glycosphingolipids that are widely distributed in the nervous system (1). Once the immune system responds to gangliosides as microbial mimics, immune-mediated neuropathies develop. Characteristic antiganglioside antibodies in peripheral blood mark several GBS variants. In acute motor axonal neuropathy (AMAN), antibodies bind to GM1 and GD1a gangliosides in the pathogenesis of nerve injuries (2–5). In contrast, anti-GQ1b antibodies are associated with the Miller Fisher syndrome (6). In addition to disease correlations, these autoantibodies against axonal targets are indicators of GBS severity (7).

### Biomarkers of GBS

Beyond antiganglioside antibodies that featured GBS, a growing number of molecules are potential biomarkers of GBS (8), including infection trigger-associated surface molecules (e.g., lipo-oligosaccharides of *Campylobacter jejuni*), active components of immune systems (e.g., FcγR/FcRL gene polymorphism, cytokines, complements, chemokines), brain-derived proteins (e.g., total protein, albumin), and neuronal composition (e.g., neurofilaments) (9–11). These biomarkers target different critical points of pathogenesis and neuronal damage in GBS.

Blood cytokines reflect elicitation of autoimmunity and disease severity in systemic autoimmune diseases, such as interleukin 6 (IL-6) and IL-10 in systemic lupus erythematosus (12). In immune-mediated neurological disorders, blood cytokines also emerge to be potential biomarkers, such as the B-cell–activating factor (BAFF) in myasthenia gravis with anti–acetylcholine receptor antibody (13, 14) and chronic inflammatory demyelinating polyneuropathy (CIDP) (15, 16). When comparing GBS patients with healthy controls, specific blood cytokines increase, including the tumor necrosis factor α (TNF-α), IL-1β, IL-6, IL-4, IL-17, and interferon γ (17). In this study, we hypothesized that blood cytokines of different parts of immune systems could reflect immune activation in GBS and patients' response to treatment. We followed a group of GBS patients during treatment and used a multiplex quantitative cytokine assay to measure their plasma cytokine changes before and after treatment. The selected cytokines represent the center of the neuroinflammation of GBS. A member of the TNF family, BAFF, appears for survival of antibody-producing B cells (18). The thymus and activation regulated chemokine (TARC), also known as CCL17, represent helper T cell 2 (T_H_2)–induced T-cell chemotaxis (19). The CD40 ligand (CD40L), also known as CD154, expresses mainly on activated CD4^+^ T cells, binds to the CD40 on B cells and antigen-presenting cells, and stands for cosimulation of B and T cells (20). C5 and C5a components (C5/C5a) are activated fragments of the complement system (21). Finally, the brain-derived neurotrophic factor (BDNF) measures the neuronal survival responses to GBS-related nerve injuries (22). By measuring these representative cytokines, we aimed to understand GBS pathogenesis and pathophysiology to identify potential biomarker(s).

## Materials and Methods

### Patient Enrollment

The patients with GBS were enrolled from June 2018 to December 2019 in the Chang Gung Memorial Hospital, Keelung City, Taiwan. The enrolled patients understood and agreed to join the study and signed written informed consent before having the first (before-treatment) peripheral blood sampling, antiganglioside antibody detection, and cytokine measurement. We excluded those patients without complete before–after blood cytokine sampling. Besides, clinicians arranged cerebrospinal fluid (CSF) studies for biochemistry analyses based on their clinical judgment. This study was approved by the institutional review board of Chang Gung Medical Foundation, with approval number 201700701A3.

### Ganglion Glycosphingolipid (Ganglioside) Antibody Detection

We performed ganglioside antibody detection on the EUROLINE platform manufactured by the EUROIMMUN (Lübeck, Germany). Samples were prepared by mixing 30 μL of plasma in 1.5 mL of 1:10 diluted sample buffer. Diluted samples were incubated with testing strips precoated with ganglioside antigens GM1, GM2, GM3, GD1a, GD1b, GT1b, and GQ1b. After incubation, the strips were washed to remove extra uncoated samples and then incubated with the enzyme conjugate, which was alkaline phosphate–labeled anti–human immunoglobulin G (IgG) and IgM (goat) to detect antiganglioside IgG and IgM in the sample, respectively. Another washing step removed the secondary antibodies. Next, the strips were incubated with the substrate, nitro blue tetrazolium chloride/5-bromo-4-chloro-3-indolyl phosphate (BNT/BCIP). The strips were air-dried and evaluated by the EUROLINE semiquantitative software.

### Quantification of the Cytokines by Fluorescent Bead–Based Multiplexing Immunoassay

The Luminex assay (Magnetic Luminex Assay: Human Premixed Multi-Analyte Kit; R&D Systems, Minneapolis, MN, USA) was a bead-based multiplexing immunoassay. Using fluorescent flow cytometry technique, the Luminex quantified multiple targets in one sample (23, 24). The plasma from GBS patients was mixed with the cytokine-specific capture antibodies coated on magnetic microparticles. In this study, the magnetic microparticle cocktail contained five kinds of precoated particles with capture antibodies against BDNF, C5/C5a, CD40L, TARC, and BAFF. The cytokine-capturing magnetic particles were mixed with the secondary detection antibody cocktail to form antibody–antigen–antibody complexes. Later, the embedded fluorophores bound to streptavidin–phycoerythrin conjugate and then excited by lasers. Finally, the Luminex analyzer followed the mechanism of flow cytometry to sort magnetic microparticle mixtures and quantified each cytokine independently. Each sample was repeated for three measurements.

## Results

### Participants and Clinical Course

Among 10 patients who met the diagnostic criteria of GBS (25), five of them completed before and after treatment cytokine testing. Their clinical scenarios are listed below and summarized in Table 1.

**Table 1:** Clinical and laboratory studies of the enrolled patients.

	**Case 1**	**Case 2**	**Case 3**	**Case 4**	**Case 5**
**Basic information**					
Age	28	16	49	71	57
Sex	Female	Female	Female	Male	Male
Medical history	CN III palsy	None	Type 2 DM	RA, HTN	NMO, SS
**Clinical presentations**					
Diagnosis	AIDP	AIDP	AIDP	MFS	AMSAN and myelitis
Onset	Subacute	Subacute	Acute	Acute	Acute
Symptoms	Dysarthria, dysphagia	Ataxic gait, limbs and facial numbness, and right CN VII palsy	Four limb weakness, ascending numbness, left CN VI and bil CN VII palsy, dysphagia, dysarthria, dysautonomia	Cerebellar ataxia, four-limb ascending numbness, bil CN III, IV, and VI palsy	Acute descending numbness below T5 level; subsequent ascending numbness
Prodrome	None	None	None	URI	None
Cancer association	None	None	None	Prostate cancer	None
Treatment	DFPP, steroid	IVIG, steroid	DFPP	DFPP, steroid	DFPP, AZA, steroid
Outcome	Good	Good	Partial	Good	Partial
**Clinical studies**					
NCS/EMG	Dem, M	Dem-Ax, M	F-wave absent	Ax, S-M	Ax, S-M
Spine MRI	n/a	Normal	Normal	n/a	T3-5 myelitis
Brain MRI	Normal	n/a	Normal	WMH	Pontine myelinolysis
CSF [protein (mg/dL)/WBCs (per μL)]	39.7/0	101.2/10	181.3/0	29.9/0	128.7/190 (Lym 83/Mo 16/Neu 1)
Paraprotein in CSF	None	None	None	IgA-lambda	None
**Autoantibody**					
Antiganglioside ab	GM1 IgM	GM2 IgM	GQ1b IgG	GQ1b IgG	GM1 IgM
Paraneoplastic ab	None	n/a	None	Yo	n/a
Other abs	None	None	None	None	AQP4, SSA
**Plasma cytokine test**					
From onset to first test	6 days	5 days	8 days	–	6 days
Days between tests	96 days	7 days	9 days	–	52 days
Treatment before the first blood test	None	None	None	–	Methylprednisolone 1,000 mg/day

*NCS/EMG, nerve conduction study and electromyography; MRI, magnetic resonance imaging; CSF, cerebrospinal fluid; WBCs, white blood cells; CN, cranial nerve; DM, diabetes mellitus; RA, rheumatoid arthritis; HTN, hypertension; NMO, neuromyelitis optica; SS, Sjögren syndrome; AIDP, acute inflammatory demyelinating polyradiculoneuropathy; MFS, Miller Fisher syndrome; AMSAN, acute motor–sensory axonal neuropathy; Bil, bilateral; URI, upper respiratory tract infection; DFPP, double-filtration plasmapheresis; IVIG, intravenous immunoglobulin; AZA; azathioprine. Dem, demyelinating; Ax, axonal; M, motor; S-M, sensorimotor; n/a, not available; WMH, white matter hyperintensity; Lym, lymphocyte; Mo, monocyte; Neu, neutrophil; ab, antibody*.

#### Case 1

A 28-year-old woman, a carrier of hepatitis B, presented with acute onset of dysphagia and dysarthria for 1 week. She came to our hospital, where the neurological examination found a decrease of bilateral gag reflex. The nerve conduction studies revealed generalized demyelinating polyneuropathy with conduction block, prolonged F-wave, and decreased amplitude. A CSF study showed white blood cells (WBCs) of 0/μL and total protein of 39.7 mg/dL. Antiganglioside antibody testing found GM1 IgM in her blood. Under the diagnosis of acute inflammatory demyelinating polyradiculoneuropathy (AIDP), she received one cycle (five sessions) of double-filtration plasmapheresis (DFPP) plus oral corticosteroid and recovered completely.

#### Case 2

A 16-year-old girl was admitted to the hospital for subacute-onset ataxic gait, four-limb and facial numbness, and right cranial nerve (CN) VII palsy. Nerve conduction studies showed generalized, mixed type with demyelinating predominant motor neuropathy. Albuminocytological dissociation of CSF study (WBCs 10/μL, total protein 101 mg/dL) and GM2 IgM antibodies in blood supported the diagnosis of AIDP. After intravenous immunoglobulin (IVIG) and steroid treatment, she had total recovery.

#### Case 3

A 50-year-old woman with type 2 diabetes mellitus presented to our hospital for weakness and ascending numbness over four limbs, left CN VI and bilateral CN VII palsy, dysphagia, dysarthria, and dysautonomia for 4 days. Brain and spine magnetic resonance imaging (MRI) showed no peculiar finding. F-wave was absent in nerve conduction studies. The sensory-evoked potential study suggested a generalized sensory conduction defect at peripheral levels. Blood antiganglioside antibody test was positive for anti-GQ1b antibody. After DFFP for AIDP, her recovery was partial, with sequelae of limb weakness and numbness.

#### Case 4

A 70-year-old man with a history of rheumatoid arthritis, hypertension, glaucoma, and traumatic subdural hemorrhage presented to our neurologic department for acute-onset cerebellar ataxia, four-limb ascending numbness, and palsies of bilateral CN III, IV, and VI. The results of the MRI of the brain were normal. Nerve conduction studies showed a pattern of chronic generalized axonal sensorimotor peripheral neuropathy. However, progressive eye movement limitations developed in the following 2 weeks. CSF study found WBCs of 0/μL and a total protein of 29.9 mg/dL. Anti-GQ1b antibody and anti-Yo antibodies were positive in his blood. Cancer surveillance found prostate cancer. Under the impression of Miller Fisher syndrome superimposed on paraneoplastic cerebellar degeneration, the patient received steroid pulse therapy and two courses of plasmapheresis. After that, his ataxia and CN palsies improved well.

#### Case 5

A 57-year-old man with a history of neuromyelitis optica (NMO) and Sjögren syndrome was admitted for recurrent myelitis presenting as acute descending numbness below T5 level for 1 week. However, subsequently ascending numbness from feet to thigh developed 1 month after the acute myelitis. A nerve conduction study revealed generalized axonal-type sensorimotor polyneuropathy. In addition to anti-aquaporin4 (AQP4) and anti-SSA antibodies, we also found GM1 IgM antibodies in his blood. He was diagnosed with acute motor–sensory axonal neuropathy (AMSAN) and recurrent myelitis of NMO. After treatment of DFFP, steroid, and azathioprine, his symptoms partially recovered, leaving him with numbness and weakness of the lower limbs.

All the enrolled patients were positive for antiganglioside antibodies, including two for GM1 IgM, one for GM2 IgM, and two for GQ1b IgG autoantibodies (Table 1). Specific antiganglioside antibodies are associated with certain GBS variants, such as the anti-GQ1b antibody's relationship to Miller Fisher variant and GBS with ophthalmoplegia (26), as our cases 3 and 4. Additional antibodies detected in the patients' blood included anti-Yo antibody in the patient with prostate cancer (case 4) and anti-AQP4 antibody and anti-Ro/SSA antibody in the patient with NMO and Sjögren syndrome (case 5) (Table 1).

### Luminex Cytokine Quantification

Notably, case 4 was apparently a case of paraneoplastic GBS because of the newly diagnosed malignancy with high cancer activity and paraneoplastic anti-Yo antibody in his blood. Therefore, to avoid heterogeneity of studying group, case 4 was excluded from the following Luminex cytokine quantification.

**Figure 1:**
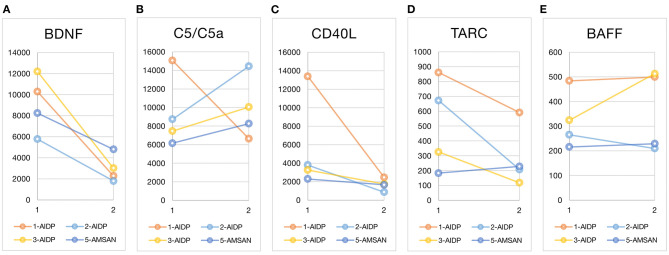
Before and after treatment plasma cytokine changes. Luminex multiplexing assay measured the five cytokines at two time points: at GBS diagnosis (before treatment) and after immunotherapy. The line graphs marked the before–after cytokine changes of each patient (the x-axis time point 1 for before treatment and 2 for after treatment). The unit of cytokines was μg/mL. The case number and type of GBS variants are listed in the graphic legend. Case 4 was excluded from the cytokine comparisons because of the obvious paraneoplastic nature of GBS in this case and the heterogeneity from other nonparaneoplastic GBS. BDNF, brain-derived neurotrophic factor; C5/C5a, the activated complement component C5 and C5a; CD40L, CD40 ligand; TARC, thymus and activation regulated chemokine; BAFF, B-cell–activating factor; AIDP, acute inflammatory demyelinating polyradiculoneuropathy; AMSAN, acute motor-sensory axonal neuropathy.

Figure 1 shows the plasma cytokine levels before and after treatment of the four nonparaneoplastic GBS patients (cases 1, 2, 3, and 5). BDNF level decreased after treatment in all these patients. The before–after change could be up to fourfold in some patients (Figure 1A). The activated complement C5/C5a increased significantly in case 2 and increased slightly in the other two cases (cases 3 and 5) but decreased in case 1 (Figure 1B). The level of soluble form CD40L was initially high in case 1 and dropped after treatment, whereas the slope of CD40L decrease was less steep in cases 2 and 3 (Figure 1C). TARC concentration ranged from 100 to 900 μg/mL in our patients; TARC showed a downward trend at different levels in cases 1, 2, and 3 after treatment (Figure 1D). The plasma level of BAFF ranged between 300 and 500 μg/mL; testing sensitivity under low concentration circumstances limited the interpretation of BAFF's interval changes (Figure 1E).

The interval of the first symptom to the first blood cytokine test ranged between 5 and 8 days (median of 6 days). The days between the two cytokine tests ranged from 7 to 96 days (median, 30.5 days), depending on the patient's condition to reach clinical stabilization to have the after-treatment blood sampling. Three of the four patients were not exposed to immunotherapy before blood sampling. One patient (case 5) had started steroid pulse therapy (methylprednisolone 1,000 mg/day) before the first blood sampling (Table 1).

## Discussion

### Summary

We reported four GBS patients and compared their plasma cytokine levels before and after immunotherapy. Using the Luminex multiplexing assay, we reduced the required amount of blood sample and measured multiple cytokines simultaneously. Two of the measured molecules, the BDNF (27) and the activated complement C5 (28, 29), have been deemed potential biomarkers or therapeutic targets for GBS. In contrast, the changes of plasma BAFF, CD40L, and TARC in GBS was reported for the first time.

The before–after change of BDNF was relatively consistent in our patients, and the potential of BDNF as an acute phase biomarker of GBS warranted large replication studies to confirm. Interindividual variations and interval changes of C5/C5a, CD40L, TARC, and BAFF level could be related to inconsistent disease phases at blood sampling, different triggers, immune responses, and interpersonal variations of responses to immunotherapy. Still, we could see a trend of early elevation of CD40L and TARC level and later elevation of C5/C5a level. Case 2 was the case that showed higher CD40L and TARC levels than other cases, which might be because the patient had previously encountered autoimmune neuropathy with CN III palsy and, for this episode, had a more robust cytokine response in the second-time confronting.

### Complement Activation in GBS

Of notice, GBS has complicated immune mechanisms during disease progression, which involve infection-induced immune mimicking, antiganglioside antibody–mediated immune reaction, imbalanced T-cell activity, and macrophage infiltration. First, antiganglioside antibodies are considered the mediators of complement activation (30). The complements activated by the antiganglioside autoantibodies lead to the formation of membrane attack complex, disrupt expression of sodium channel, result in conduction block, and then exhibit the clinical signs of nerve damage (31). Some *in vitro* studies supported the concept that C3b and C5b-9 had harmful effects on peripheral nerves (8, 32). Complement-activated deposition of C3b on the outer surface of Schwann cells can lead to the initiation of vesiculation of myelin. Infiltration of activated macrophages and T cells follows the myelin break and subsequently induces axonal degeneration (33, 34). A serial observation found that complements kept aggregating around nerves where the blood–nerve barrier was broken and led to nerve injury during the first 4 weeks of GBS (28). We also observed a delayed elevation of complement active components C5/C5a. The relatively high level of C5/C5a did not appear at the initial stage but at a later stage in most of our patients (cases 2, 3, and 5). Therefore, the complement-mediated nerve injury did not quickly cease and might be the reason for persistent limb weakness or numbness.

### T-Cell Immunity in GBS

Different groups of T cells participate in the pathogenesis of GBS. CD4^+^ helper T cell dysregulation goes through the entire disease course of GBS. At the initial phase of GBS, T_H_1 proinflammatory activity is upregulated. In the later stage, the upregulation of the T_H_2 anti-inflammatory cytokine replaces the T_H_1 cytokine activity (35). Together with the T_H_1 cells, circulating T_H_17 and T_H_22 cells are also significantly increased in GBS patients, correlated with disease severity, and downregulated in response to IVIG treatment (36). Regulatory T (Treg) cell is another group of T cells that critically mediates the autoimmunity of GBS. Temporarily reducing of circulating Treg is related to the loss of its negative regulations on immune response in GBS (37, 38). Augmentation of Treg rescued nerve injuries in the experimental autoimmune neuritis (EAN) animal model (39). On the contrary, CD8^+^ cytotoxic T cells increase in peripheral blood (40) and infiltrate endoneurium, especially in those patients with a subacute clinical course of GBS (28). To summarize, imbalanced T-cell function is crucial for the development of GBS. Antagonistic effects among the T_H_1, T_H_2, T_H_17, T_H_22, and Treg cells determine the development, progression, or recovery of GBS (41).

In our patients, plasma TARC and CD40L levels initially elevated and later dropped in some patients (cases 1, 2, and 3) but kept unchanged at a low level in the other one (case 5, Figure 1). Although the inconsistency might represent interindividual differences of T-cell activation, the type of GBS variant might matter. In a study of lymphocyte subset, the AIDP group showed significantly higher percentages of CD4^+^CD45RO^+^ memory T cells and lower percentage of CD4^+^CD45RA^+^ naive T cells than the healthy control; this ratio reversed after IVIG treatment. However, the AMAN variant did not possess this disparity to the healthy control or the before–after difference (42). The significant before–after changes of TARC and CD40L in our AIDP patients (cases 1, 2, and 3) might also reflect the T-cell involvement in AIDP type but not in other variants (case 5).

### Costimulatory Molecules in GBS

Costimulatory molecules increase in number and enhance the cellular immune responses in several autoimmune diseases, such as systemic lupus erythematosus, rheumatoid arthritis, type 1 diabetes mellitus, and multiple sclerosis (43); using monoclonal antibodies targeting costimulatory molecules is one of the developing treatments of autoimmune diseases (44). The CD40 and CD40L are a pair of costimulatory molecules between B cells, macrophage, dendritic cells, and activated T cells; upregulation of CD40 appears together with the increase of plasmacytoid dendritic cells in the acute phase of GBS patients (45). Also, in the animal model of GBS, CD40 is essential in creating EAN in mice (46); the dramatically increased expression of CD40 and CD40L marks the cooperation of B and T cells in the initiation of neuritis (47). Although enhanced expression of other costimulatory molecules has already been shown in GBS, such as the CD80 and CD86 (i.e., the B7-1 and B7-2 costimulatory molecules) (48) and the inducible T-cell costimulator (49), the CD40L was first shown in our report to be involved in pathogenesis and be a potential biomarker in the acute phase of GBS.

### Chemokines in GBS

Trafficking inflammatory cells across the blood–nerve barrier is crucial in developing GBS; chemokines and chemokine receptors express in the endoneurium of peripheral nerves and circulate in the blood of GBS patients and EAN animal models (50). Previous studies of GBS and EAN have identified several chemokines and their receptors as pathogenic marks, including CCL2-CCR2 (51) and CXCL10-CXCR3 (52). Some others were considered treatment targets, although some succeed (such as CCR2) (53) and some failed (like CCR5) (54). Except the aforementioned chemokines, the CCR4 family is the other potential pathogenic target of GBS; positive staining of CCR4 was shown in the sural nerve biopsy of AIDP patients and was localized on invading T cells (55). The importance of CCR4 and its two ligands, TARC (CCL17) and CCL22, has also been noticed in central nervous system autoimmunity and studied in the experimental autoimmune encephalomyelitis murine model (56). To our knowledge, this is the first report that identified TARC (CCL17) as a potential biomarker of acute GBS, and the results warranted replication and animal model confirmation.

### B-Cell Immunity in GBS

In previous studies, the B cells seem not at the center of GBS pathogenesis. The peripheral blood B-cell subset did not alter in GBS (42). However, increase in memory B-cell ratio in GBS patients with IgG antiganglioside antibodies suggested the antibody-initiated immune chain reaction (57). In our study, only case 3 had a before–after change of BAFF concentration, but not significant (Figure 1). Of notice, case 3 was positive for IgG antiganglioside antibodies, whereas the other cases were positive for IgM antiganglioside antibodies (Table 1). Although we could not confirm the differences of B-cell subsets between IgG- and IgM-related GBS, the slight increase of BAFF level in our patients might echo the importance of B-cell immunity in IgG-related GBS. Similarly, BAFF plays a key role in CIDP and determines if the patient responds to IVIG (15, 16).

In addition to that, antiganglioside antibodies are pathogenic in GBS (58, 59). Gangliosides are widely distributed on the outer leaflets of plasma membranes of various tissues but particularly abundant in neuronal cells. The sialic acids and negative charge of gangliosides make them form a protective shield to avoid autologous immunity and against pathogen attachment. Antiganglioside antibodies break this protective shield and allow complements to attach to neuronal cells easily and further cause massive cell injury. In addition, the deposition of antiganglioside antibodies forms the immune complexes, which cause inflammation and tissue damage, trigger leukocyte recruitment, augment antigen presentation, and activate the complement system. Furthermore, antiganglioside antibody–induced membrane structural changes alter the normal neuronal function that relies on the intact neuronal membrane (1, 60). Therefore, the B-cell immunity remains important in GBS regarding the pathogenic features of antiganglioside antibodies.

### The Neurotrophic Factor BDNF in GBS

In our report, the BDNF level elevated consistently at the acute phase (before-treatment blood sampling) in all four cases. The member of the neurotrophin family, BDNF, involves in neuronal plasticity, survival, synaptogenesis, and neurotransmitters modulation (61). Even if BDNF is not a cytokine, increasing evidence has linked BDNF to neuroinflammation (22, 62). Although BDNF elevation signifies neuroinflammatory processes in brain disorders, its significance in peripheral nerve disorders is not fully understood.

During repairing peripheral nerve injury, the neurotrophins, particularly BDNF, serve for axon regeneration. *Via* signaling through cell surface tropomyosin receptor kinases (Trk) receptor and p75 neurotrophin receptor, two separate intracellular signaling pathways work for neuronal survival and neuronal plasticity (63). Increased expression of BDNF mRNA and TrkB mRNA in motor neurons suggests that BDNF responds to nerve injury (64). BDNF can be synthesized by dorsal root ganglion, as well in the circumstance of peripheral nerve inflammation (65). In lesioned peripheral nerves, Schwann cells dramatically increase the BDNF synthesis with a much higher amplitude than that of nerve growth factor (66).

Monoclonal antibodies are reliable in quantifying the blood concentration of BDNF (67). Many studies used BDNF level for clinical correlations or outcome predictions in various neurological and psychiatric diseases, such as Alzheimer disease (68, 69), Parkinson disease (70), Huntington disease (71), major depressive disorder (72), and multiple sclerosis (73, 74). BDNF augmentation was considered a potential disease-modifying strategy in neurodegenerative (75) and neuroinflammatory diseases (76). In inflammatory neuropathies, subcutaneous injection of BDNF had been tried on GBS patients to improve recovery (27); however, the results did not support its therapeutic use because of the small sample size, nonsignificant effects on improving disability after 4 weeks [mean difference, 0.75; 95% confidence interval, −1.14 to 2.64; very low certainty of the evidence (77)], and early termination of the trial. Although not being considered as a therapeutic agent, BDNF remains potential as a biomarker of the acute phase of GBS and warrants further studies.

### Does Immunotherapy Affect Cytokine Levels?

Therapeutic apheresis, including plasma exchange and plasmapheresis, is an effective treatment of GBS (78). Plasma exchange is a centrifugation-based technique to separate patients' blood components and replace them with fluid and plasma from healthy people. Plasmapheresis separates patients' plasma *via* a filtration-based device to remove large molecules such as antibodies and immune complexes and infuses the filtrated plasma back to the patients (79). Plasma exchange is theoretically able to remove more small molecules, such as cytokines, than plasmaspheres and results in a short-term benefit in improving the disability score of GBS; however, their long-term benefits to GBS patients do not differ (80). Currently, the argument of whether plasma exchange or plasmapheresis alters circulating cytokines remains inconclusive (81). Presumably, the intensity of plasma removal might matter, and only intensive plasma removal correlates with significant cytokine changes (82). The post–plasma exchange cytokine rebounding phenomenon might be another factor of the inconsistent results (83). Moreover, the mechanisms of action of plasma exchange and plasmapheresis are far more complex than merely removal of blood components. They may involve in the proliferation of normal B-cell population, correction of the imbalanced T_H_1/T_H_2 antagonism, and upregulation of suppressor T and Treg cells (79). Therefore, the cytokine changes we measured are the overall effects after therapeutic apheresis.

IVIG is another equally effective treatment of GBS (84). IVIG is suggested to achieve therapeutic effects in GBS *via* reduction of IL-1, intercellular adhesion molecule-1, and especially TNF-α, which is significantly higher in GBS than other neurological disease controls (85–87). The complex reaction after IVIG infusion also regards the increase of T-cell production of transforming growth factor β and upregulation of Fcγ receptor on B cells and monocytes (87).

Glucocorticoids strongly repress the immunomodulatory transcription factors, nuclear factor κB (NF-κB) and activator protein-1, to achieve therapeutic effects in autoimmune diseases (88, 89). The depression of NF-κB results in multiple immunosuppressing responses, including downregulation of proinflammatory cytokines, chemokine, and adhesion molecules and reduction of inflammatory T cells and macrophages (90). Circulating anti-inflammatory cytokines also increase in response to glucocorticoids (91).

To summarize, plasmapheresis, IVIG, and steroid treatment in our patients all may more or less affect their plasma cytokine levels *via* multiple immune mechanisms. The cytokine changes we measured after treatment are the net effects of disease recovery and treatment-related immune corrections. Therefore, overall considerations of GBS pathogenesis and immunotherapy effects and observations of multiple targets of immune systems are necessary for interpreting the before–after changes of cytokine levels.

### Limitations of the Study

There were several limitations to this study. First, the small case number restricted its generalization to all GBS patients. The power of discussion on each single GBS variant or IgM/IG-related GBS cases was limited. Second, lacking a control group limited the statistical power of this study and restricted the generalizability of the results. Even if the before–after paired comparison is advantageous in highlighting the interindividual difference of immune responses, comparisons to a proper control group could objectively evaluate the value of these biomarkers.

Third, the interval between two blood sampling was not consistent among the patients. Although we arranged the second blood sample according to stabilization of individuals' conditions, the wide range of sampling intervals might raise considerations of multifactorial interferences to cytokine levels, such as environmental factors, underlying diseases, and acute stress responses. In contrast, the short between-test period might confound the results because patients might still be in the acute phase, and cytokine levels had not reached a plateau. A reasonable and fixed sampling time will help to overcome this limitation in future studies.

Finally, we limited the cytokine tests to the five representative cytokines because of the limited experimental resources, and it might not show the complete picture of disease mechanisms. Several commonly reported crucial cytokines, such as the IL-6, IL-10, interferon γ, and TNF-α, were not measured here. Choosing a group of cytokines per immune cell type will expand our knowledge of the immune mechanisms of GBS and have good use of the strength of the Luminex platform.

## Data Availability Statement

The raw data supporting the conclusions of this article will be made available by the authors under reasonable request, without undue reservation.

## Ethics Statement

The studies involving human participants were reviewed and approved by the institutional review board of Chang Gung Medical Foundation, with approval number 201700701A3. Written informed consent to participate in this study was provided by the participants or the participants' legal guardian/next of kin.

## Author Contributions

C-LW contributed to study conception, data analysis, and revision of the manuscript. C-HC drafted the manuscript. S-WL, Y-YC, W-YH, W-CW, and F-CS contributed to study design and data acquisition. Y-CW draft and revised the manuscript, collected data, and run laboratory analysis and interpretation. All authors have read and approved the manuscript.

## Conflict of Interest

The authors declare that the research was conducted in the absence of any commercial or financial relationships that could be construed as a potential conflict of interest.

## Publisher's Note

All claims expressed in this article are solely those of the authors and do not necessarily represent those of their affiliated organizations, or those of the publisher, the editors and the reviewers. Any product that may be evaluated in this article, or claim that may be made by its manufacturer, is not guaranteed or endorsed by the publisher.
